# Chronotype distribution and clinical correlates in adults diagnosed with ADHD

**DOI:** 10.1192/j.eurpsy.2026.10558

**Published:** 2026-07-31

**Authors:** B. A. Oroian, P. Nechita, A. Szalontay

**Affiliations:** 1 ”Grigore T. Popa” University of Medicine and Pharmacy; 2 ”Socola” Institute of Psychiatry, Iasi, Romania

## Abstract

**Introduction:**

Circadian rhythm disturbances are increasingly recognized as relevant clinical features in adults with Attention-Deficit/Hyperactivity Disorder (ADHD). Chronotype, defined as an individual’s biologically driven preference for timing of sleep and activity (morningness vs eveningness), has been associated with ADHD symptom severity, executive dysfunction, and emotional regulation. Despite growing interest in the interplay between chronotype and psychiatric conditions, data remain limited in adult ADHD populations

**Objectives:**

This study aimed to assess the distribution of chronotypes among adults diagnosed with ADHD according to ICD-10 criteria and to examine associations between chronotype, symptom severity, and sleep quality.

**Methods:**

A total of 50 adults (aged 18–45 years) diagnosed with ADHD based on ICD-10 criteria were recruited through outpatient psychiatric services as part of a cross-sectional observational study. Participants completed the Morningness–Eveningness Questionnaire (MEQ) to determine chronotype. ADHD symptom severity was evaluated using the Adult ADHD Self-Report Scale (ASRS), and sleep quality was measured with the Pittsburgh Sleep Quality Index (PSQI). Statistical analyses included chi-square tests for distribution and Pearson correlations to explore associations between chronotype, symptom burden, and sleep quality.

**Results:**

Among 50 adults with ADHD, 64% were classified as evening chronotypes, 24% as intermediate, and 12% as morning types, based on MEQ scores. Evening-type individuals showed significantly higher ADHD symptom severity, particularly inattention (ASRS: 25.3 vs. 19.1 and 17.6), and reported later sleep onset (mean: 00:23 AM) and longer sleep latency (42.7 min). Sleep quality, measured by the PSQI, was poorest in evening types (mean: 9.8), with more frequent insomnia and non-restorative sleep. Functionally, evening chronotype was associated with greater daytime dysfunction, including difficulty initiating tasks, lower energy levels during standard work hours, and higher rates of reported emotional dysregulation, particularly irritability and low frustration tolerance. Correlational analysis showed a moderate negative association between morningness (MEQ score) and ADHD severity and sleep quality. Chronotype independently predicted inattention severity and sleep quality after adjusting for age and sex. No associations were found with comorbid anxiety or depression.

**Conclusions:**

Evening chronotype is overrepresented in adults with ADHD and is associated with greater symptom severity and sleep dysfunction. Chronotype assessment may offer valuable insights for tailoring behavioral and pharmacological interventions, especially regarding sleep hygiene and treatment timing. Incorporating circadian rhythm evaluation into routine clinical practice may enhance diagnostic precision and patient outcomes in ADHD management.

**Disclosure of Interest:**

None Declared

